# Interference of metamizole in the measurement of serum creatinine concentration

**DOI:** 10.1515/almed-2023-0163

**Published:** 2024-04-26

**Authors:** María-José Castro-Castro, Isabel Cachón-Suárez, Andrea Maestre-Fernández, Rosa Navarro-Badal

**Affiliations:** Biochemistry Core, Laboratori Clínic Territorial Metropolitana Sud, Hospital Universitari de Bellvitge, Barcelona, Spain; Biochemistry and Physiology Department, Universitat de Barcelona, Barcelona, Spain

**Keywords:** metamizole interference, creatinine enzymatic method; creatinine interference

## Abstract

**Objectives:**

There is little literature on the interference of metamizole in measurement creatinine concentration by the enzymatic method. Some studies have postulated that the dipyrone molecule is responsible for interfering in the last reaction of the enzymatic method sequence, due to its similarity with the 4-aminophenazone molecule. The aim of this study is to examine the interference of the presence of metamizole in the measurement of serum creatinine concentration by the enzymatic method.

**Methods:**

An interference study of the measurement of creatinine concentration was carried out with two measurement procedures (enzymatic and Jaffe), by adding different concentrations of metamizole to pool from 30 serum samples of patients.

**Results:**

The interference study indicates that the results of serum creatinine concentrations in patients, as measured by the enzymatic method, decrease with the addition of increasing concentrations of metamizole.

**Conclusions:**

There is interference with metamizole in the measurement of serum creatinine concentration by the enzymatic method that it is have not seen in the Jaffe method.

## Introduction

Serum creatinine is one of the most commonly measured products in clinical chemistry laboratories worldwide [1]. Until recently, the widely used method for creatinine measurement was the Jaffe method, which involves the reaction of creatinine with picric acid at alkaline pH. Although simple and inexpensive, Jaffe methodology is nonspecific because alkaline picrate can react with any compound containing a methylene group. Several modifications have been made to reduce interference in the Jaffe method. However, unfortunately, kinetic modifications have not completely resolved specificity issues [2, 3]. Enzymatic estimation of creatinine was first described in 1937 and is inherently more specific because of the selectivity of enzymes [2]. The Jaffe assays are more susceptible to interfering substances than the enzymatic method in the frequency and the degree of interference. However, enzymatic assays are not immune to non-specificity [4]. For one of the enzymatic method reagents that is currently marketed by Roche Diagnostics, the manufacturer declares some interferences in its specifications, with rifampicin, levodopa, calcium dobesilate, methyldopa, N-ethylglycine, dl-proline, 2-phenyl-1,3-indandion (phenindion), dicynone (etamsylate) and metamizole, among others [5].

The frequency of errors produced by analytical interference is not well known. However, the literature shows that errors produced in the analytical phase represent between 7 and 13 % of all errors [6]. There is little literature on the interference by metamizole described by Roche Diagnostics in creatinine concentration by the enzymatic method. According to Bagnoud and Reymond, the methyl-amino-antipyrine metabolite appears to be responsible for the interference. Methyl-amino-antipyrine is the active substance, since metamizole is a prodrug [7].

Some studies have postulated mechanisms of interference with analytical methods by metamizole or other drugs [8, 9]. It is possible that the dipyrone molecule is responsible for interfering in the last reaction of the enzymatic method sequence, due to its similarity with the 4-aminophenazone molecule.

Previous *in vitro* studies showed that metamizole interferes with the Jaffe method and the enzymatic method, at different concentrations of metamizole (subtherapeutic, therapeutic, or toxic concentrations) [10]. A study by Bojko et al. demonstrated that intravenous administration of metamizole interfered with the measurement of creatinine concentration by the enzymatic method [11]. However, another study showed that metamizole did not interfere with the Randox enzymatic method of measuring creatinine concentration [12].

Other drug interferences have been described. They, have in common interference in the Trinder reaction (wich is responsible for the last step of the reaction for the measurement of creatinine by the enzymatic method) [9].

Around 21 % of inpatients in Bellvitge University Hospital (L’Hospitalet de Llobregat, Barcelona, Spain) are subjected to metamizole treatment with intravenous administration and 18 % to oral administration, due to its wide use as an analgesic. Therefore, it is important to determine how this drug interferes in the measurements of biochemical magnitudes in the clinical laboratory.

The aim of this study is to examine whether metamizole interferes in the measurement of serum creatinine concentration by the enzymatic method.

## Materials and methods

Creatinine concentration was measured in a Cobas c702 analyzer (Roche Diagnostics, Rotkreuz, Switzerland). The analytical performance of the two procedures for measuring serum creatinine concentration was studied: the enzymatic method and the Jaffe method. The mean coefficient of analytical variation and systematic error during the study for both methods were calculated using two levels of internal quality control: Levels 1 and 3 of Liquid Assayed Multiqual control materials (references 694 and 696, lot 45,890), manufactured by Bio-Rad (Madrid, Spain), were processed for 16 days. The coefficient of variation and the systematic error were estimated, taking as the true value the mean of the group values ​​with the same measurement method in the accumulated data from Bio-Rad’s External Quality Assurance Program.

An interference study of the measurement of creatinine concentration has been carried out with the two measurement procedures (enzymatic and Jaffe), by adding different concentrations of metamizole to a serum pool to obtain mixing of 30 serum samples from patients with an average concentration of 126 μmol/L. This concentration is not far from the population reference value. The range of metamizole concentrations that was studied includes the maximum therapeutic concentrations (Cmax) reported for endovenous administration in some pharmacokinetic studies: 0.03 [13], 0.05–0.1 g/L [14].

To prepare the different dilutions of metamizole, 2 g/5 mL (400 g/L) bottles of metamizole were used, and solutions of 100, 20, 10, 5, 2.5, 1.25, 0.63, 0.31 and 0.16 g/L were obtained by adding sodium chloride. A total of 20 µL of each metamizole solution was added to different aliquots of the serum pool of 180 µL each. In addition, 20 µL of sodium chloride was added to 180 µL of the serum pool as a negative control. The Wilcoxon test was performed using the medians to compare the results obtained in the two measurement procedures. A p-value<0.05 was considered statistically significant.

The difference in percentage of serum creatinine concentration was calculated for each dilution, with reference to the control samples. These differences were compared with the allowable total error established by our laboratory, based on the state of the art (20 %) [15].

Six serum samples from patients that were suspected of being contaminated with intravenous metamizole administration were stored at −20 °C. The suspicion of interference was due to the fact that in the results validation process it was found that the result in these samples was very different from the previous result of the same patient. In addition, the change limits (the deltacheck) system applied in the clinical laboratory gave an alarm to carry out the review of the results (25 % decrease in the change limit and 40 % increase in the change limit).

These samples were from inpatients in the hospital from various medical departments (emergency, intensive medicine, palliative care, cardiology and gastroenterology). The samples were thawed and processed using the two measurement procedures (enzymatic and Jaffe).

Personal data relating to patients obtained through the present study were processed in accordance with Regulation (UE) 2016/679 of the European Parliament on Data Protection. In addition, the study complied with the ethical principles for medical research involving human subjects adopted in the Declaration of Helsinki of the World Medical Association. The Research Ethics Committee of Bellvitge University Hospital approved this paper for its publication (reference number PR240/22).

Statistical analyses were performed using SPSS software version 17.0 and Microsoft Excel 2010.

## Results

The coefficient of variation and systematic error were calculated, and enzymatic and Jaffe methods conformed to the laboratory’s established quality specifications, which required 7.4 % for imprecision and 8.8 % for systematic error. As such, it was ensured that both methods meet the quality requirements established in the laboratory.

**Figure 1:**
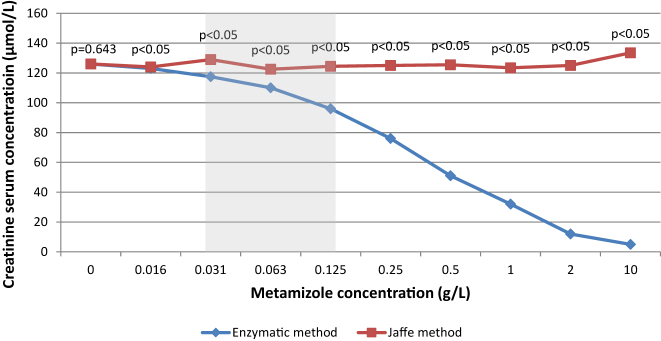
Graphic with results of serum creatinine concentrations from serum pool samples from patients by the two measurement procedures (enzymatic and Jaffe) with the addition of different concentrations of metamizole. p: p-value of Wilcoxon test. Grey zone: metamizole therapeutic concentration.

In Figure 1, we present a graphical representation of serum creatinine concentrations from the serum pool using two measurement procedures (enzymatic and Jaffe) with varying concentrations of metamizole. The p-value (p) for the Wicoxon test is indicated, and the grey zone signifies the therapeutic concentration of metamizole for intravenous concentration. The graph illustrates a decrease in creatinine concentration with the enzymatic method as metamizole concentration increases, which contrasts with the Jaffe method where no such decrease is observed.

Furthermore, Table 1 shows the medians of serum creatinine concentrations with an interquartile range and percentage differences for each dilution, with reference to the control samples. The grey zone indicates the therapeutic concentration of metamizole for intravenous concentration. An increase in metamizole concentration leads to a decrease in serum creatinine concentration and an elevated percentage of variation compared to the condition without metamizole addition, as observed in the enzymatic method. In contrast, the Jaffe method does not exhibit this variation.

**Table 1:** Comparison of creatinine concentration medians in serum pool using enzymatic and Jaffe methods by adding increasing concentrations of metamizole.

Metamizole concentration, g/L	Creatinine concentration with enzymatic method Median (IQR), µmol/L (%)	Creatinine concentration with Jaffe method Median (IQR), µmol/L (%)
0	126 (123, 130) (0.0 %)	126 (124, 130) (0.0 %)
0.016	123 (123, 130) (2.4 %)	124 (122, 127) (1.6 %)
0.031	118 (110, 121) (6.7 %)	129 (127, 132) (2.4 %)
0.063	110 (108, 113) (12.7 %)	123 (122, 126) (2.8 %)
0.125	96 (93, 100) (23.8 %)	125 (123, 128) (1.2 %)
0.25	76 (74.78) (39.7 %)	125 (123, 129) (0.8 %)
0.5	51 (49.52) (59.5 %)	126 (124, 130) (0.4 %)
1	33 (32.34) (74.6 %)	124 (123, 126) (2.0 %)
2	12 (11.12) (90.5 %)	125 (123–126) (0.8 %)
10	5 (5.6) (96 %)	134 (133, 135) (6.0 %)

Grey zone, metamizole therapeutic concentration. %, percentage of variation respect no metamizol addition. IQR, interquartile range (Q1, Q3).

Table 2 presents creatinine concentration results in serum samples that were suspected of contamination due to intravenous metamizole administration, analyzed by both methods. In some processed samples (samples 1, 3, 5 and 6), a significant variation in creatinine concentration is observed between the enzymatic and Jaffe methods. The concentration measured by the enzymatic method was consistently lower than that obtained with the Jaffe method.

**Table 2:** Creatinine concentrations in serum samples on suspicion of being contaminated with intravenous metamizole administration obtained by enzymatic and Jaffe methods.

Serum sample	Creatinine concentration with enzymatic method, µmol/L	Creatinine concentration with Jaffe method, µmol/L
Sample 1	15	63
Sample 2	42	41
Sample 3	8	93
Sample 4	27	28
Sample 5	3	110
Sample 6	2	141

## Discussion

The graph (Figure 1), which compares the results of serum creatinine concentration obtained by the enzymatic and Jaffe methods, highlights a distinct trend: as the concentration of metamizole increases, the interference effect becomes more pronounced in the enzymatic method. Conversely, the Jaffe method shows no such interference with increasing metamizole concentrations.

This observation strongly indicates the occurrence of interference when the enzymatic method is used in the presence of metamizole, while the Jaffe method seems unaffected by this interference. In fact, the interference may lead to creatinine concentration results falling below the measurement range.

Moreover, the Wilcoxon test produced a significant result (p<0.05) for all dilutions except the control (absence of metamizole), which confirmed the substantial differences between the two methods. This finding unequivocally confirms that the enzymatic method is sensitive to the interference caused by metamizole when its concentrations exceed 0.016 g/L in the sample, while the Jaffe method remains unaffected.

When the percentages of serum creatinine were compared with the total allowable error, according to our results, samples with metamizole concentrations up to 0.062 g/L were analytically relevant.

In accordance with pharmacokinetic studies [13], maximum metamizole concentrations in plasma following intravenous administration range from 0.03 to 0.015 g/L after 1 h and 0.15 g/L after 2 h of oral administration (endovenous and oral doses of 1,000 mg). It is conceivable that elevated serum concentrations could arise from direct venous access, which potentially cause contamination due to intravenous administration. Importantly, our study is limited by the absence of serial measurements following intravenous metamizole administration.

In Table 2, a significant discrepancy in creatinine concentration values is apparent between samples 1, 3, 5, and 6 as measured by the enzymatic and Jaffe methods. This variance suggests that metamizole may interfere with the enzymatic method, which would potentially influence processes associated with the Trinder reaction [8, 9].

Our results are consistent with specific studies [10, 11] that suggest interference from metamizole in an enzymatic creatinine measurement, although they diverge from observations related to the Jaffe method. While Luna-Zaizar et al. [10] reported positive interference, our study did not demonstrate any interference in Jaffe method.

Our study recognizes the exclusion of metamizole’s main metabolites from analysis due to measurement limitations, which represents a noteworthy constraint in our study. The ability to identify samples that are susceptible to interference would be enhanced if these metabolite concentrations were measurable. Nonetheless, our results indicate that patients receiving intravenous metamizole may produce falsely decreased creatinine concentrations.

In our study, it was not possible to conduct the analysis of interference at different concentrations of creatinine. Future studies could address this limitation identified in our work.

Recognizing samples that are susceptible to this interference presents challenges in routine laboratory practice. Suspicion typically arises during result validation, particularly when a notable concentration decrease is noted compared to a previous results for the same patient. Consequently, reaching out to the blood drawing service to inquire about any recent intravenous metamizole administration becomes crucial. These steps are basic in identifying interference, and if detected, obtaining a new patient sample becomes imperative to avoid inaccurate serum creatinine results.

While sustaining both enzymatic and Jaffe methods, might not be feasible for many clinical laboratories, the Jaffe method, recognized for its freedom from metamizole interference, could serve as a confirmatory tool for potential interference. Nevertheless, a more pragmatic approach involves requesting a new patient sample in instances of suspected interference and improving pre-analytical sampling processes to mitigate the risk of intravenous contamination.

Nevertheless, the precise intravenous metamizole dose that initiates interference remains unknown, underscoring the need for *in vivo* studies involving serial sampling after metamizole administration. The laboratory should disseminate guidelines to the hospital’s nursing staff, emphasizing the significance of blood extraction preceding metamizole administration.

In conclusion, interference from metamizole is apparent in serum creatinine measurements when employing the enzymatic method but absent in the Jaffe method. Therefore, to confirm whether a sample has mistakenly lowered its creatinine concentration using the enzymatic method, reprocessing with the Jaffe method may be undertaken. The interference observed with metamizole in serum creatinine measurement likely pertains to processes specific to enzymatic creatinine measurement, contrasting with the interference-free nature of the Jaffe methods.
